# Oncogenic Osteomalacia as a Harbinger of Recurrent Osteosarcoma

**DOI:** 10.1080/13577149977712

**Published:** 1999-09

**Authors:** Elizabeth B. Lamont, Melissa K. Cavaghan, Bruce E. Brockstein

**Affiliations:** 1 Section of Hematology-Oncology Department of Medicine University of Chicago 5841 S. Maryland Avenue, MC2115 Chicago IL 60637 USA; 2 Section of Endocr inology Department of Medicine University of Chicago Chicago IL 60637 USA

## Abstract

*Discussion.* Oncogenic osteomalacia is a rare paraneoplastic syndrome of skeletal demineralization from renal phosphate loss. Patients with this disorder have the characteristic clinical, laboratory, and radiographic findings of hyperphosphaturic osteomalacia. Although the pathophysiology has not yet been clearly delineated, a humoral factor produced by the tumor is suspected to be the cause.

*Purpose.* We report the first case of oncogenic osteomalacia that improved with chemotherapy, discuss this paraneoplastic syndrome, and review the medical literature regarding its etiology.


*Sarcoma* (1999) **3**, 95–99

ORIGINAL ARTICLE

### Oncogenic osteomalacia as a harbinger of recurrent osteosarcoma

ELIZABETH B. LAMONT,[1] MELISSA K. CAVAGHAN[2] & BRUCE E. BROCKSTEIN[1]

[1]*Section of Hematology-Oncology and* [2]*Section of Endocrinology, Department of Medicine, University of Chicago, Chicago, IL 60637, USA*

**Abstract**

*Discussion*. Oncogenic osteomalacia is a rare paraneoplastic syndrome of skeletal demineralization from renal phosphate loss. Patients with this disorder have the characteristic clinical, laboratory, and radiographic findings of hyperphosphaturic osteomalacia. Although the pathophysiology has not yet been clearly delineated, a humoral factor produced by the tumor is suspected to be the cause.

*Purpose*. We report the first case of oncogenic osteomalacia that improved with chemotherapy, discuss this paraneoplastic syndrome, and review the medical literature regarding its etiology.

**Key words**: *oncogenic osteomalacia, etiology, chemotherapy*

### Introduction

With fewer than 100 cases reported in the medical literature, oncogenic osteomalacia is a rare endocrinological paraneoplastic syndrome characterized by defective bone mineralization from renal phosphate loss. Tumor elaboration of a phosphaturic factor is the putative mechanism. Although occasionally reported in other tumor types, oncogenic osteomalacia is almost exclusively described in patients with benign tumors of mesenchymal origin. Characteristically, patients present with signs and symptoms of osteomalacia, i.e., waddling gait, joint deformities, bone pain, muscle weakness, anorexia, fatigue and occasionally long bone fractures. Laboratory examination is notable for hypophosphatemia in the setting of inappropriate phosphaturia, but usually normal PTH levels. 1,25-Vitamin D is inappropriately normal relative to the coexisting hypophosphatemia and is frankly depressed in many cases. Evaluation with plain films usually reveals osteopenia, and occasionally the pseudofractures (Looser zones) and subperiosteal erosions seen in osteomalacia. Bone scans also show the characteristic findings of osteomalacia, which is uptake that is diffuse and/or focal. Because the tumors associated with this syndrome are typically quite small ($\leq$ 1 cm), the clinical symptoms often precede the identification of the tumor. In an otherwise healthy patient who presents with osteomalacia due to hyperphosphaturia, evaluation for an occult tumor should be considered. For patients with known tumors, surgical resection usually cures them of the syndrome.

### Case presentation

CB is a 68-year-old Caucasian woman diagnosed in September 1993 with stage II B osteosarcoma of the left femur. She was treated with neoadjuvant cisplatin, adriamycin and ifosfamide, followed by a limb-sparing resection revealing nearly 100% tumor necrosis. Post-operatively, she began adjuvant chemotherapy, but this treatment was terminated in August 1994 after only one cycle of carboplatin because of persistent difficulties with wound healing and declining performance status. She resumed a vigorous lifestyle including a 60-h work week and daily exercise. She was well until May 1996 when she developed a single pulmonary metastasis which was then surgically resected. After a normal routine tumor surveillance CT scan of the chest and upper abdomen in January 1997, she presented in February 1997 to her oncologist complaining of painful lower extremity edema and bilateral ankle pain. Her examination was notable for 2+ bilateral tender pitting edema, tender ankles, but no stigmata of heart failure, hepatic failure, nephrotic syndrome, or deep venous thromboses. Additionally, she had painless proximal interphalangeal joint swelling and significant ulnar deformity of her metacarpal phalangeal joints. Laboratory studies revealed significant hypophosphatemia (1.5 mg/dl; normal range, 2.6–4.4), borderline hypocalcemia (8.3 mg/dl; normal

Correspondence to: Elizabeth Lamont, University of Chicago, 5841 S. Maryland Avenue, MC2115, Chicago, IL 60637, USA. Tel: +1 773 702 4143; Fax: +1 773 702 3163; E-mail: elamont@medicine.bsd.uchicago.edu

1357-714X print/1369-1643 online/99/020095-05 © 1999 Taylor & Francis Ltd range, 8.1–10.2), and a mildly elevated alkaline phosphatase (200 U/l; normal range, 51–153). Of note, her serum sodium (136 meq/l; normal range, 134–149), bicarbonate (25 meq/l; normal range, 23–30), potassium (4.1 mg/dl; normal range, 3.5–5.2), magnesium (2.2 meq/l; normal range, 1.6–2.5), and creatinine (0.5 mg/dl; normal range, 0.5–1.4) were normal. Additionally, both her parathyroid hormone (PTH) and 1,25$(OH)_2$-vitamin D levels were normal (57 pg/ml; normal range, <60; and 27 pg/ml; normal range, 15–60, respectively). Plain films of the ankles revealed osteopenia, and a bone scan (see Fig. 1) revealed symmetrical uptake in the joints of the hands, feet and spine. Her clinical symptoms and hypophosphatemia were relatively refractory to high doses of oral phosphate (500 mg elemental phosphorus daily) but her edema improved with lasix (20 mg daily).

In March, the patient was seen at the Endocrinology Clinic where a 24-h urine revealed both inappropriate phosphaturia (777 mg; normal range, 500–1500) and calciuria (262 mg; normal range, 100–200) given her serum values. Additionally, there was mild glycosuria (1.05 g; normal range, 0.0–0.5), mild proteinuria (0.15 g; normal range, 0.05–0.1), and trace elevation of urinary glycine. No other amino acids were detected. Notably, a review of the patient's extensive laboratory evaluation prior to and after surgery, and chemotherapy at her initial diagnosis of osteosarcoma in 1993, revealed no similar abnormalities. Bone densitometry of her lumbar spine (L2–L4) and her femoral neck revealed severe osteoporosis. The diagnosis of osteomalacia was assumed and, given her sarcoma history, the etiologies considered were oncogenic osteomalacia or, less likely, a renal tubular

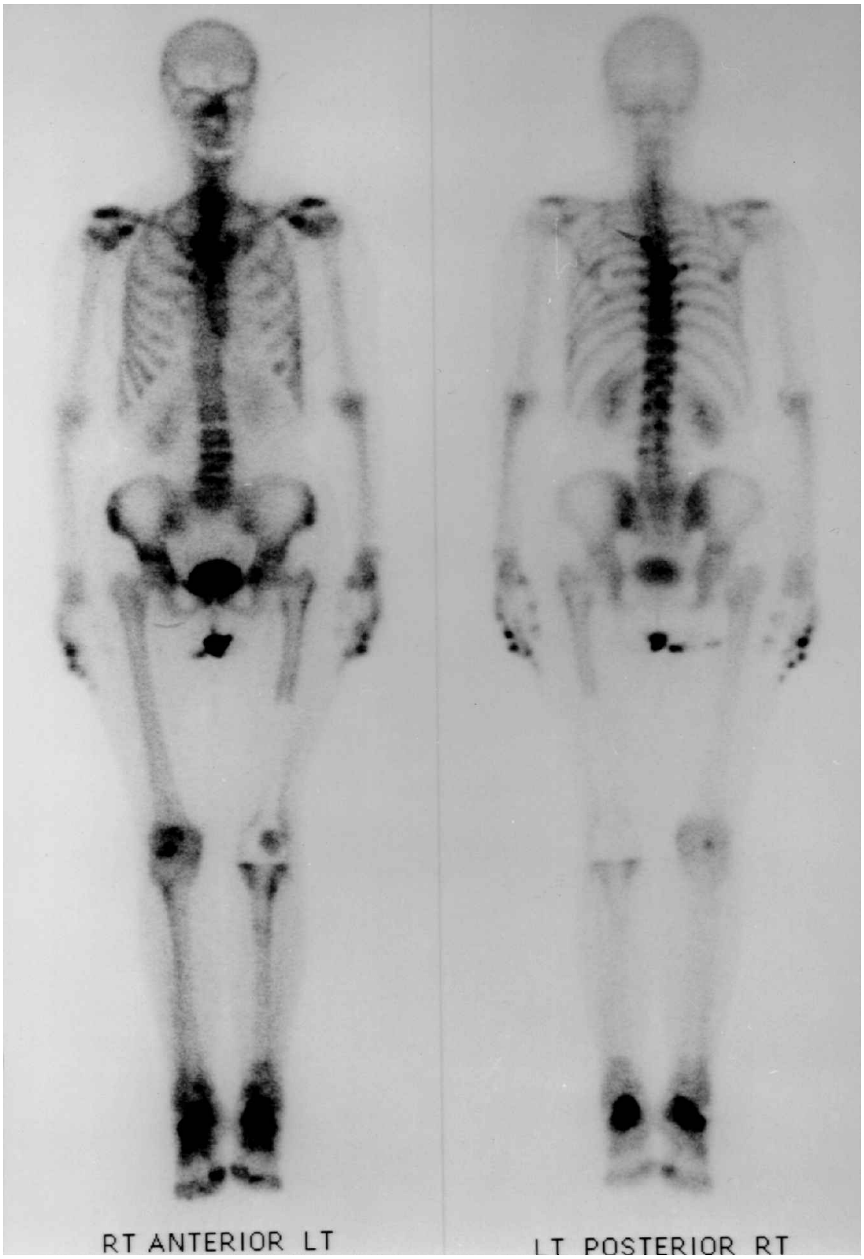

**Fig. 1.** *The whole body bone scan of our patient with oncogenic osteomalacia reveals the multi-focal radiotracer avidity of osteomalacia.*

defect (e.g., Fanconi syndrome or chronic toxicity) from prior chemotherapy (ifosfamide). The patient's course was not consistent with the Fanconi syndrome, primarily because her hypophosphatemia was temporally distant from ifosfamide treatment. Further, her aminoaciduria was negligible, and she had no evidence of potassium, magnesium or bicarbonate wasting that would accompany a more generalized proximal tubular defect. The patient's course was also not consistent with a chronic tubulopathy from ifosfamide, since her severe phosphate wasting was out of proportion to other evidence of proximal tubulopathy and was unresponsive to phosphate repletion, uncharacteristic of previously described ifosfamide-induced renal toxicity.[1] While some degree of chronic tubular damage from prior ifosfamide and cisplatin was almost certain, ifosfamide toxicity alone was felt unlikely to explain the acute onset and refractory hypophosphatemia.

Given the working diagnosis of oncogenic osteomalacia, the endocrinology consultants recommended evaluation for possible recurrent tumor, increased her dose of phosphate supplement to 1500 mg of elemental phosphorous daily and added vitamin D (800 U daily) and calcium carbonate (1500 mg daily). Her hypophosphatemia did not improve and a chest CT obtained in May revealed a left hilar mass and a single large liver lesion, both suspicious for recurrent or a second malignancy. In June multiple liver lesions and an obstructing pancreatic head lesion were noted, and biopsies of both locations were consistent with metastatic osteosarcoma. Within 2 weeks of a single dose of adriamycin* (30 mg/m$^2$) given in July 1997, the patient's serum phosphate increased into the normal range for the first time since diagnosis of the syndrome 6 months previously. However, the normalization was fleeting as it declined within the subsequent 2 weeks. Later that month the patient developed thoracic spinal cord compression and expired in August 1997 while enrolled in a home hospice. Table 1 contains a timeline of her disease, electrolyte values and medical interventions. While the patient's mean phosphate level during her period of osteomalacia (preceding the dose of adriamycin) was 1.76 mg/dl, the mean phosphate level after the single dose of adriamycin until death was 3.82 mg/dl. A post-adriamycin 24-h urine for phosphate was not collected.

### Discussion

Osteomalacia is a disorder of defective mineralization of osteoid, organic bone matrix. Since osteoid is mineralized to bone through deposition of crystals composed of calcium and phosphate, prolonged deficiency of phosphate, calcium, or both, can beget osteomalacia. Chronic hypophosphatemia results from inadequate intake or excessive loss and presents in inherited or acquired forms. Inadequate phosphate intake can be the result of extensive intestinal disease, the presence of phosphate binders in the diet, or secondary to vitamin D deficiency (due to low dietary intake, lack of sun exposure, renal or hepatic disease, and inherited disorders of vitamin D metabolism). Excessive urinary loss due to renal tubular dysfunction can be inherited (e.g., autosomal recessive and X-linked hypophosphatemic rickets, Fanconi syndrome) or acquired (e.g., renal tubular acidosis, toxic damage by heavy metals or certain chemotherapeutic agents, and tumor-induced osteomalacia). Chronic hypocalcemia as a result of intestinal disease, abnormalities of vitamin D and parathyroid hormone, or systemic acidosis is an unusual cause of osteomalacia since symptoms of hypocalcemia often result in earlier medical attention and are only rarely seen in hypoparathyroidism and pseudohypoparathyroidism.

There are several hypotheses for the mechanism of urinary phosphate loss in oncogenic osteomalacia, with a humoral mechanism by far the most compelling based on clinical observations and experimental data. The clinical observations that support a humoral mechanism are that the culprit tumors are often small and remote from the kidney, that tumor removal results in both rapid resolution of phosphaturia and in rapid skeletal remineralization, and that some culprit tumors appear to have neurosecretory granules on EM suggesting peptide hormone production.[2,3] *In vivo* and *in vitro* experiments suggest the presence of a transmittable phosphaturic factor. Investigators report phosphaturia in dogs and rats injected with tumor extracts[4–6] and in athymic mice transplanted with tumor.[7] In single cell assays, investigators have described inhibition of both renal tubular sodium-dependent phosphate transport[8,9] and renal tubular 25(OH)D-1$\alpha$-hydroxylase when media from tumor cell cultures were added to cultures of animal renal tubule cells.[7] These experiments resonate with the clinical findings of phosphaturia and decreased 1,25(OH)$_2$-vitamin D in patients with these neoplasms. However, whether the circulating factor has PTH-like activity is still unclear: tumor extracts have stimulated cAMP in some cases[8,10] but not in others.[4,9]

The mainstay of management of oncogenic osteomalacia has been removal of tumor through complete surgical resection. Since the condition is usually associated with localized benign tumors and not metastatic malignant disease, this approach is usually successful. Unfortunately, our patient had metastatic malignant disease that was not resectable. However, the fact that tumor-specific chemotherapy was effective in at least transiently improving her hypophosphatemia may support the theory that oncogenic osteomalacia is paraneoplastic in origin. However, why the patient's tumor progressed while her phosphate was still in the normal range is not clear. It

* Dose of adriamycin reduced from 60 mg/m$^2$ because of hyperbilirubinemia.

**Table 1.** *A dramatic, but transient improvement in osteomalacia indices after a single dose of adriamycin in a patient with oncogenic osteomalacia from an osteosarcoma.*

| Event | Dx primary tumor | Neoadjuvant chemotx | Surgery | | Pulmonary relapse, f/b resection | | Clinically evident osteomalacia | | | CT: lung, & liver mets | | Adriamycin 30 mg/m$^2$ x1 | | | | Dx SCC | | Death |
|---|---|---|---|---|---|---|---|---|---|---|---|---|---|---|---|---|---|---|
| Lab Test | 10/5/93 | 1/25/94 | 6/14/94 | 5/13/96 | 8/20/96 | 10/28/96 | 1/27/97 | 2/10/97* | 4/15/97 | 5/24/97 | 6/16/97 | 7/1/97 | 7/15/97 | 7/21/97 | 7/23/97 | 7/24/97 | 7/26/97 | 8/28/97 |
| Phosphate | 3.9 | 3.7 | 3.5 | 4.4 | 3.5 | 4.0 | 1.8 | 1.5 | 1.5 | 1.7 | 2.0 | | **5.3** | **3.7** | **3.2** | **4.6** | **2.3** | |
| Calcium | 9.1 | 8.8 | 9.0 | 9.0 | 9.0 | 8.7 | 8.9 | 8.3 | 8.2 | 8.1 | 6.5 | | 5.7 | 6.2 | 6.8 | 7.3 | 6.8 | |
| Alkphos | 184 | 139 | 145 | 109 | 104 | 126 | 162 | 200 | | 1188 | | | 362 | 228 | 378 | 204 | | |
| Potassium | 4.2 | 4.0 | 3.8 | 3.9 | 3.7 | 3.4 | 3.3 | 4.1 | 4.1 | 4.3 | 2.8 | | 2.1 | 3.7 | 4.1 | 3.9 | 4.3 | |
| Creatinine | 0.9 | 0.8 | 0.9 | 0.9 | 0.7 | 0.7 | 0.7 | 0.5 | | | 0.7 | | 1.0 | 0.6 | 0.8 | 0.6 | 0.8 | |

* Daily oral Phosphate (500 meq QD), potassium, and Lasix intiated. On 3/17/97, daily oral phosphate increased (1500 meq QD) and calcium carbonate (1500 mg QD) and vitamin D (800 U QD) added to pre-existing regimen and continued until her admission for spinal cord compression (SCC) (7/24/97) when the vitamins and minerals were discontinued.

may be that the kinetics of the putative paraneoplastic phosphaturic factor and the kinetics of the tumor are not equal.

### Conclusion

We report an atypical case of the rare entity oncogenic osteomalacia. The case was atypical because it was associated with a malignant tumor, because the syndrome presented only with a second recurrence of the tumor, not with its initial presentation or first relapse, and because it transiently improved with chemotherapy. Like others with this paraneoplastic syndrome, our patient had characteristic clinical, laboratory and radiographic findings of hyperphosphaturic osteomalacia. While the pathophysiology of oncogenic osteomalacia is still not entirely clear, a paraneoplastic etiology has been proposed. Through our report of a patient's transient resolution of hypophosphatemia coincident with chemotherapy, we provide further evidence to suggest that this syndrome may be paraneoplastic in origin.

### References

1 Rossi R, Godde A, Kleinebrand A, *et al.* Unilateral nephrectomy and cisplatin as risk factors of ifosfamide-induced nephrotoxicity: analysis of 120 patients. *J Clin Oncol* 1994; 12:159–65.

2 Stone MD, Quincey C, Hosking DJ. A neuroendocrine cause of oncogenic osteomalacia. *J Pathol* 1992; 167:181–5.

3 Wilkins GE, Granleese S, Hegele RG, *et al.* Oncogenic osteomalacia: evidence for a humoral phosphaturic factor. *J Clin Endocrinol Metab* 1995; 80(5):1628–34.

4 Aschinberg LC, Solomon LM, Zeis PM, *et al.* Vitamin D-resistant rickets associated with epidermal nevus syndrome: demonstration of a phosphaturic substance in the dermal lesions. *J Pediatr* 1977; 91:56–60.

5 Popotvtzer MM. Tumor-induced hypophosphatemic osteomalacia: evidence for a phosphaturic cyclic AMP-independent action of tumor extract. *Clin Res* 1981; 29:418A. abstract.

6 Lau K, Stom MC, Goldberg M, *et al.* Evidence of a humoral phosphaturic factor in oncogenic hypophosphatemic osteomalacia. *Clin Res* 1979; 27:421A (Abstract)

7 Cai Qiang, Hodgson SF, Kao PC, *et al.* Brief report: inhibition of renal phosphate transport by a tumor product in a patient with oncogenic osteomalacia. *New Engl J Med* 1994; 330(23):1645–9.

8 Nelson AE, Namkung HJ, Patava J, *et al.* Characteristics of tumor cell bioactivity in oncogenic osteomalacia. *Mol Cell Endocrinol* 1996; 124:17–23.

9 Miyauchi A, Fukase M, Tsutsumi M, Fujita T. Hemangiopericytoma-induced osteomalacia: tumor transplantation in nude mice causes hypophosphatemia and tumor extracts inhibit renal 25-hydroxyvitamin D1-hydroxylase activity. *J Clin Endocrinol Metab* 1988; 67:46–53.

10 Seshadri MS, Cornish CJ, Mason RS, Posen S. Parathyroid hormone like bioactivity in tumours from patients with oncogenic osteomalacia. *Clin Endocrinol* 1985; 23:689–97.
